# hsa_circ_0006459 and hsa_circ_0015962 affect prognosis of Dengue fever

**DOI:** 10.1038/s41598-019-55153-3

**Published:** 2019-12-19

**Authors:** JinHua He, Yue Ming, Yu MinLi, ZePing Han, JianWei Jiang, JiaBin Zhou, BoYing Dai, YuBing Lv, Meng Ling He, Mao Fang, YuGuang Li

**Affiliations:** 1 Department of Laboratory Medicine, Central Hospital of Panyu District, Guangzhou, Guangdong 511400 P.R. China; 2 Infection Department, Central Hospital of Panyu District, Guangzhou, Guangdong 511400 P.R. China; 3 Department of Biochemistry, Medical College, Jinan University, Guangzhou, Guangdong 510630 P.R. China; 4 Medical Laboratory of Shenzhen Luohu People’s Hospital, Shen zhen, Guangdong 518001 P.R. China; 5 Department of Pathology, School of Basic Medical Science, Guangzhou Medical University, Guangzhou, Guangdong 511436 P.R. China

**Keywords:** Dengue virus, Molecular biology

## Abstract

Circular ribonucleic acids (circRNAs) are widely expressed in human cells and play an important role in the pathogenesis of many diseases. Some circRNAs have microRNA (miRNA) binding response elements and interact with miRNA to regulate the expression of target genes.Four patients with a preliminary diagnosis of dengue fever (DF), peripheral whole blood sample in anticoagulant was collected before treatment (pretreatment group) and after effective treatment (posttreatment group), and eight samples were separated and used to screen differentially expressed circRNAs with microarray analysis. The relative expression level of circRNAs was determined using reverse-transcription polymerase chain reaction (RT-PCR). TargetScan v7.1 and miRDB v5 bioinformatics software were used to predict circRNA-binding miRNAs; dual luciferase reporters were constructed to detect binding between circRNA and miRNA. Microarray screening revealed 263 differentially expressed circRNAs in peripheral leukocytes pretreatment versus posttreatment; 107 of these were upregulated and 156 were downregulated. RT-PCR confirmed that hsa_circ_0015962 was significantly upregulated and hsa_circ_0006459 significantly downregulated (*P* < 0.05). Moreover, hsa_circ_0015962 binds to miR-4683, and hsa_circ_0006459 binds to miR-133b.Downregulation of hsa_circ_0006459 and upregulation of hsa_circ_0015962 affect the treatment response of DF and are potential biomarkers in DF patients. The molecular mechanism involves hsa_circ_0006459-mediated targeted negative regulation of miR-133b and hsa_circ_0015962-mediated targeted negative regulation of miR-4683.

## Introduction

Circular ribonucleic acids (circRNAs), a group of newly discovered endogenous non-coding RNAs (ncRNAs), are the latest hot topic in RNA research. Unlike linear RNAs containing a 5′ cap structure and a 3′ adenylate tail, circRNAs form a special covalent closed-loop structure with no 5′ to 3′ direction or polyadenylate tail^1^. Studies have shown that circRNAs, a group of endogenous RNAs widely found in mammalian cells, regulate gene expression at the post-transcriptional level^2^. CircRNAs are widely expressed in human cells and play an important role in the pathogenesis of various tumors^3^. In-depth study of circRNA expression and regulation contributes to the prevention and diagnosis of related diseases. Some circRNAs harbor microRNA (miRNA)-binding response elements and interact with miRNA to regulate the expression of target genes^4^.

Dengue fever (DF) is an acute infectious disease caused by dengue virus (DENV) transmission through the bite of infected *Aedes aegypti* mosquitoes. It is one of the most prevalent insect-borne zoonotic viral infections worldwide and is particularly prevalent in cities and suburban areas in tropical and subtropical regions. The incidence of DF has been rising substantially worldwide over the past decades. Clinical manifestations include acute onset, sudden high-grade fever, apparent fatigue, loss of appetite, and nausea, which are often accompanied by severe headache, pain around the eyes, general muscle pain, and bone and joint pain, with or without conjunctival hyperemia and facial, neck, and chest redness^5^. Leukopenia, especially neutropenia, is the primary laboratory finding and often occurs in the early stage of DF. White blood cells (WBC) count usually starts to decrease from day 2 and reaches its nadir (as low as 2 × 10^9^/L) by days 4–5. Moreover, thrombocytopenia occurs in most cases, and platelet count can be as low as <10 × 10^9^/L^6^, with varying degrees of liver and myocardial damage.

The role of circRNA in the pathogenesis and treatment response of DF is unknown. In this study, we collected peripheral blood samples from 43 DF patients pre- and post treatment, analyzed the relative expression of differentially expressed circRNAs, and investigated potential molecular mechanisms, in order to provide a basis for further research on the pathogenesis of DF and related mechanisms of treatment response.

## Methods

### Case information

Case selection: A total of 43 DF patients diagnosed and treated at the Panyu District, Central Hospital of Guangzhou City between November 1, 2017 and February 28, 2018 were included in this study. with informed consent and human ethics approval from the panyu central hospital of Guangzhou human research ethics committees. DF was diagnosed (clinical diagnosis or laboratory confirmation) and classified as general or severe based on criteria from ‘Dengue: Guidelines for Diagnosis, Treatment, Prevention and Control’ from the World Health Organization (WHO; 2009)^7^ and ‘The Guidelines for the Diagnosis and Treatment of Dengue Fever’ (Edition 2, 2014) from the National Health and Family Planning Commission of the People’s Republic of China^8^. Exclusion criteria were chronic liver disease, diabetes, chronic kidney disease, malignant tumor with metastasis, or hematologic disease. This study was conducted in accordance with the Declaration of Helsinki and was approved by the Ethics Committee of our hospital. According to the treatment guidelines, patients were placed on bed rest with a light diet and were quarantined in a ward with beds covered with insecticide-treated mosquito nets and other anti-mosquito equipment until more than 5 days after disease onset and body temperature had returned to normal for more than 24 h. Moreover, symptomatic and supportive care was given, and measures were taken to prevent and treat various complications

### Isolation of leukocytes

Fresh venous blood was drawn and leukocytes were isolated within 6 h after blood collection to ensure their survival. Density gradient centrifugation was employed for leukocyte isolation at 18–20 °C using Red Blood Cell Lysis Buffer (Solarbio, Beijing, China), according to the manufacturer’s instructions.

### Total RNA extraction from leukocytes and circRNA microarray analysis

Peripheral blood leukocytes were collected pre- and post-treatment from DF patients to extract total RNA using TRIzol reagent (Thermo Fischer Scientific, Waltham, MA) according to the manufacturer’s instructions. RNA samples were sent to KANGCHEN Inc. (Shanghai, China) for Arraystar circRNA Microarray analysis. The Human 8 × 15 K circRNA Array, which contains 9114 circRNA probes, is manufactured by Arraystar Technologies (Rockville, MD). Each circRNA was identified by using a specific probe that targets the specific splice junction of circRNA. Sample labeling and array hybridization were performed according to the manufacturer’s protocol (Arraystar). R software package (version 3.1.2) was used for normalizing the raw data and for subsequent data processing. Two groups of profile differences (pretreatment samples versus posttreatment samples), and the absolute fold change for each circRNA was computed^9^. The predicted interaction between circRNAs/miRNAs predicted use the targetscan and miRanda software.

### RT-PCR assays of significant differentially expressed circRNAs

RNA was extracted with TRIzol reagent and reverse-transcribed with Moloney Murine Leukemia Virus reverse transcriptase (M-MLV) into complementary Deoxyribonucleic acid (cDNA) template for PCR; each sample was run in triplicate. We use URP unified reverse primer for miRNA PCR (5′-CTCAACTGGTGTCGTGGA-3′). PCR primers are shown in Tables 1, 2. β-actin was used as the internal reference, and the 2^−ΔΔCt^ method was used to derive relative quantitative results.

**Table 1:** Gene primers.

Gene	Sequenc	Product Length (bp)
β-actin	F:5′GTGGCCGAGGACTTTGATTG3′ R:5′CCTGTAACAACGCATCTCATATT3′	73
hsa_circ_0006459	F:5′GAGAGAAGAAAATGGATACACTGC3′ R:5′AAGCACATCTTGTTGCGTAGG3′	166
hsa_circ_0015962	F:5′ATGGACATCACTGGAGAGGAAA3′ R:5′GACAAAGGTCAGACAAGGGTTC3′	128

**Table 2:** Primer for miRNA.

miRNA	RT for miRNA	miRNA F
hsa-miR-4683	CTCAACTGGTGTCGTGGAGTCGGCAATTCAGTTGAGATCGGGCG	ACACTCCAGCTGGGTGGAGATCCAGTGCTCG
hsa-miR-4692	CTCAACTGGTGTCGTGGAGTCGGCAATTCAGTTGAGATCTGATA	ACACTCCAGCTGGGTCAGGCAGTGTGGGTA
hsa-miR-4802-3	CTCAACTGGTGTCGTGGAGTCGGCAATTCAGTTGAGGCTTGAAG	ACACTCCAGCTGGGTACATGGATGGAAACCT
hsa-miR-7161-3p	CTCAACTGGTGTCGTGGAGTCGGCAATTCAGTTGAGCCTGGAGA	ACACTCCAGCTGGGTAGATCTTTGACTCTGGCAGTC
hsa-miR-6893-5p	CTCAACTGGTGTCGTGGAGTCGGCAATTCAGTTGAGGCTCCACC	ACACTCCAGCTGGGCAGGCAGGTGTAGGG
hsa-miR-598-5p	CTCAACTGGTGTCGTGGAGTCGGCAATTCAGTTGAGGCTCACAC	ACACTCCAGCTGGGGCGGTGATCCCGATGGT
hsa-miR-301a-5p	CTCAACTGGTGTCGTGGAGTCGGCAATTCAGTTGAGAGTAGTGC	ACACTCCAGCTGGGGCTCTGACTTTATTGC
hsa-miR-133a-3p	CTCAACTGGTGTCGTGGAGTCGGCAATTCAGTTGAGCAGCTGGT	ACACTCCAGCTGGGTTTGGTCCCCTTCAAC
hsa-miR-92a-2-5p	CTCAACTGGTGTCGTGGAGTCGGCAATTCAGTTGAGGTAATGCA	ACACTCCAGCTGGGGGGTGGGGATTTGTTG
hsa-miR-1297	CTCAACTGGTGTCGTGGAGTCGGCAATTCAGTTGAGCACCTGAA	ACACTCCAGCTGGGTTCAAGTAATT
hsa-miR-133b	CTCAACTGGTGTCGTGGAGTCGGCAATTCAGTTGAGTAGCTGGT	ACACTCCAGCTGGGTTTGGTCCCCTTCAAC

### Cell culture

293T cell lines were purchased from the Shanghai Institute of Cell Biology (Shanghai, China). Cells were cultured in Dulbecco’s Modified Eagle’s Medium/Nutrient Mixture F-12 Ham containing 10% fetal bovine serum, and maintained in a humidified incubator at 37 °C with 5% CO_2_. Cells in logarithmic growth with 95% viability were subjected to further experiments.

### Luciferase reporter assay

Genomic DNA was extracted from peripheral blood cells and used as a template for the linear sequence of circRNA, and *Xho*I and *Not*I restriction sites were introduced. The PCR amplification product was subjected to double digestion with the respective enzymes and cloned into the psiCHECK-2 vector. The ligated products were then transformed into *Escherichia coli* DH5a cells. A blue/white screening assay and PCR were performed to identify positive clones. Dual-luciferase assay (Promega, Madison, WI) was then performed according to the manufacturer’s instructions^10^.

### Statistical analysis

Data are expressed as the mean ± standard deviation (SD) and were processed using SPSS statistical software version 20.0 (SPSS, Chicago, IL). Statistical comparisons were performed using one-way analysis of variance.

## Results

### Clinical data of subjects

In this study, Dengue fever patient were divided into two groups based on their T-scores for hematological indices, and these differed significantly between the two groups (Table 3). Mean values for total and differential WBC count, red blood cell count, hemoglobin, hematocrit, mean corpuscular hemoglobin, platelet count (PLT), plateletcrit, and the glucose, alanine transaminase, total bile acids, and electrolyte levels are also shown. There were significant differences between the pretreatment and post treatment groups as shown in Table 3.

**Table 3:** Clinical results of Dengue fever patients in both groups.

Item	prtreatment	posttreatment	T value	P value
WBC(white blood cell)	3.38 ± 2.23	5.52 ± 2.48	−4.582	0
NEUT%(neutrophil)	51.2 ± 17.9	46.1 ± 12.3	1.447	0.155
LYMPH%(lymphocyte)	34.9 ± 14.7	36.0 ± 10.34	−0.384	0.703
MONO% (monocytes)	12.3 ± 5.3	14.5 ± 4.3	−3.225	0.002
EOS%(eosinophilia)	0.8 ± 0.63	2.8 ± 1.3	−5.855	0
EASO%(Basophil)	0.8 ± 0.60	0.6 ± 0.13	0.595	0.555
NEUT#	1.767 ± 0.02	2.698 ± 0.03	−2.289	0.027
LYMPH#	1.031 ± 0.23	1.876 ± 0.56	−5.692	0
MONO#	0.378 ± 0.052	0.761 ± 0.03	0.709	0.482
EOS#	0.027 ± 0.0005	0.153 ± 0.03	−6.06	0
EASO#	0.03 ± 0.001	0.03 ± 0.002	−0.418	0.678
RBC(red blood cell)	4.73 ± 0.96	4.50 ± 0.63	3.736	0.001
HGB(hemoglobin)	139 ± 15.63	130 ± 18.06	4.573	0
HCT(Hematocrit)	41.0 ± 3.72	39.2 ± 4.6	3.551	0.001
MCV(mean corpuscular volume)	87.0 ± 5.10	87.6 ± 5.63	−1.811	0.077
MCH(mean corpuscular hemoglobin)	29.4 ± 198	28.9 ± 1.99	4.979	0
MCHC(mean corpuscular hemoglobin concentration)	338 ± 11.63	331 ± 14.78	4.199	0
SD-RDW(Standard deviation of Red blood cell distribution width)	40.3 ± 2.66	42.5 ± 3.57	−6.314	0
CV-RDW(Coefficient of Variability-Red Blood Cell Distribution Width)	12.8 ± 0.69	13.3 ± 0.56	−1.169	0.249
PLT(Platelet)	92 ± 10.56	159 ± 23.0	−5.379	0
MPV(mean platelet volume)	11.28 ± 1.266	11.52 ± 1.16	−1.126	0.267
PCT(platelet crit)	0.108 ± 0.05	0.180 ± 0.06	−5.254	0
PDW(platelet volume distribution width)	14.35 ± 2.99	14.67 ± 3.05	−0.506	0.615
P-LCR(platelet -larger cell ratio)	35.31 ± 9.76	36.95 ± 9.29	−0.943	0.352
K(kalium)	3.55 ± 0.32	3.77 ± 0.24	−2.668	0.019
Na(Natrium)	136.4 ± 3.34	138.3 ± 2.44	−3.55	0.004
Cl(chlorine)	101.8 ± 3.58	103.3 ± 16.3	−2.375	0.034
Ca(calcium)	2.27 ± 0.69	2.21 ± 0.03	1.002	0.335
UREA(carbamide)	3.6 ± 1.51	3.1 ± 1.12	1.34	0.202
CRE(creatinine)	73 ± 9.9	80 ± 10.6	0.953	0.357
GLU (glucose)	6.07 ± 1.75	4.90 ± 0.56	2.599	0.021
CO2(Carbon dioxide)	23.2 ± 3.11	24.1 ± 3.12	−0.82	0.427
ALT(Alanine aminotransferase)	41 ± 9.6	118 ± 19.8	−2.916	0.012
AST(Aspartic aminotransferase)	77 ± 8.72	128 ± 117.62	−1.076	0.302
ALT/AST	0.6 ± 0.01	1 ± 0.3	−2.768	0.016
GGT(Glutamyl transferase)	43 ± 8.2	110 ± 11.3	−1.765	0.101
TBIL(Total bilirubin)	9.6 ± 1.9	13.0 ± 2.9	−2.258	0.045
DBIL(Direct bilirubin)	3.1 ± 1.4	4.3 ± 1.5	−1.646	0.128
IBIL(Indirect bilirubin)	6.6 ± 1.8	8.7 ± 1.5	−2.102	0.059
ALP(Alkaline phosphatase)	66 ± 10.77	64 ± 8.96	−0.637	0.537
TBA(Total bile acid)	8.5 ± 1.32	3.7 ± 1.10	2.4	0.035
LDH(Lactate dehydrogenase)	320 ± 21.3	365 ± 26.9	−1.063	0.323
CK(Creatine kinase)	266 ± 31.2	80 ± 19.6	1.393	0.206
HBDH (Hydroxybutyrate dehydrogenase)	188 ± 25.60	214 ± 53.65	−0.995	0.353
CKMB(Creatine kinase isoenzyme MB)	17 ± 7.98	15 ± 1.3	0.628	0.55
LDHI (Lactate dehydrogenase isoenzyme)	47 ± 11.2	44 ± 12.17	0.632	0.548
CRP(C-reactive protein)	9.7 ± 0.96	7.9 ± 0.87	1.752	0.14

Note: The data above are expressed as the mean ± standard deviation.

### CircRNA expression profiles

**Figure 1:**
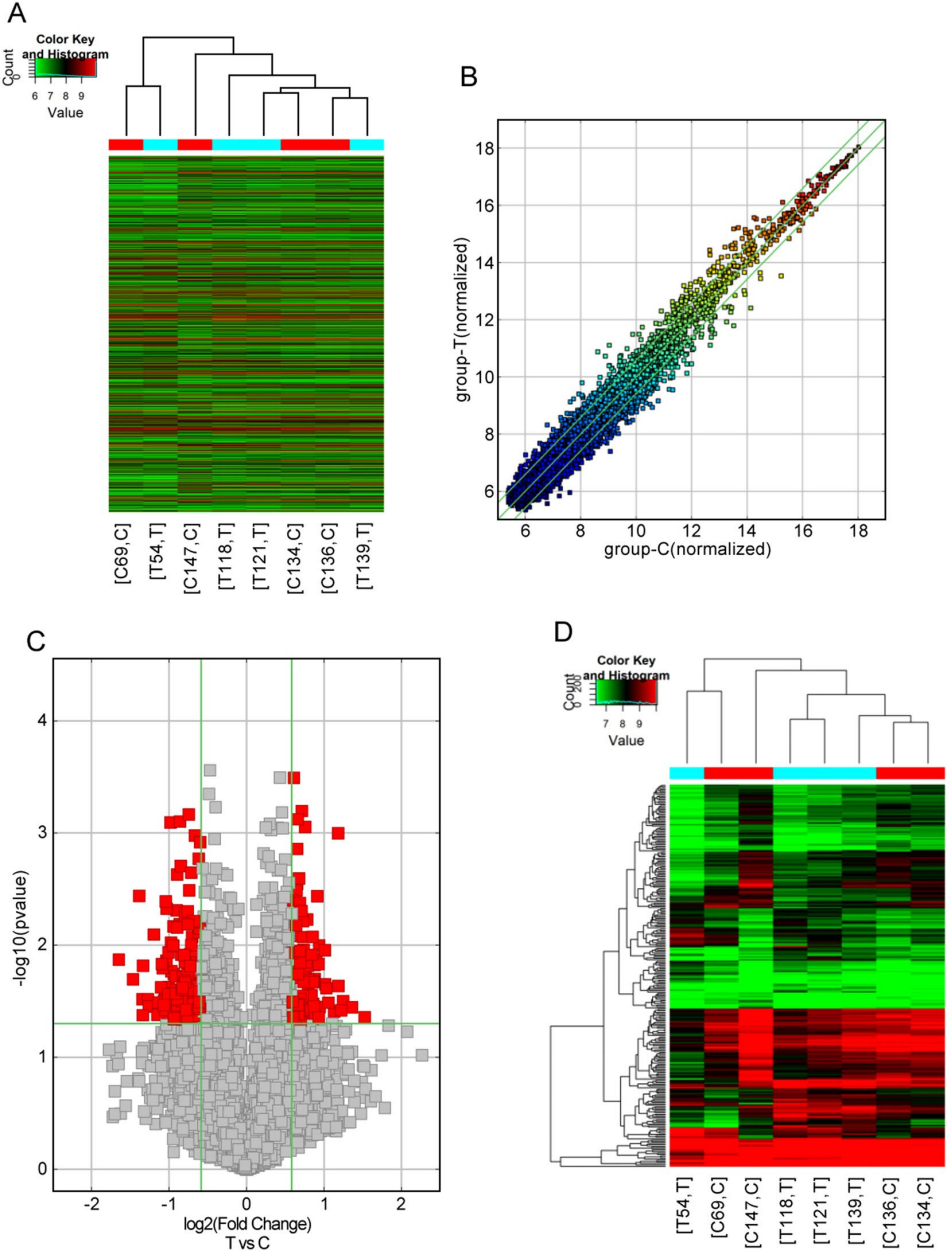
Arraystar circRNA microarray analysis of peripheral blood mononuclear cells (PMBCs) in a DF patient. (**B**) Scatter plot showing circRNA expression variation between the two samples. The X and Y axes represent normalized signal values. circRNAs above the top green line and below the bottom green line demonstrate a comparatively more than 2.0-fold change between the two samples; (**C**) Volcano plots showing differential expression of circRNAs; (**A**) Hierarchical clustering heat map showing the different circRNA expression profiling of the 8 samples; (**D**) Clustergram showing all of circRNA expression profiling of the samples.

**Figure 2:**
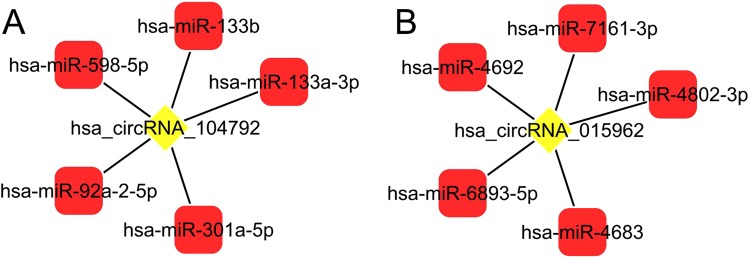
Prediction of circRNA-binding miRNAs using bioinformatics software. (**A**) hsa_circ_0006459-binding miRNAs; (**B**) hsa_circ_0015962-binding miRNAs.

In total, 10958 circRNAs were analyzed by circRNA microarray in four pairs of pretreatment and posttreatment samples from patients with a preliminary diagnosis Dengue fever. Fold changes (FC) of circRNA expression in both groups were measured to identify differentially expressed circRNAs (Fig. 1A). Volcano plots identified substantial changes in the levels of 263 circRNAs (FC ≥ 1.5), including 107 upregulated and 156 downregulated circRNAs(Supplementary File 1); this was statistically significant (P < 0.05; Fig. 1B–D). Four circRNAs were selected based on the FC of differential expression and initial expression level, including 2 upregulated circRNA and 2 downregulated circRNA (Table 4),We have performed GO and KEGG analyses(Supplementary Files 2, 3). Two circRNAs with the most change in expression were selected for sequence analysis and the potential miRNA targets of these circRNAs were predicted accordingly (Fig. 2A,B).

**Table 4:** Differential expression circRNA peripheral blood leucocyte was screened by CircRNA microarry

circRNA	chrom	circRNA_type	GeneSymbol	Expression level	P value
hsa_circ_0000931	chr19	Sense overlap	ZNF573	up	0.0635
hsa_circ_0015962	chr1	exonic	RNPEP	up	0.0236
hsa_circ_0006459	chr9	exonic	GNAQ	down	0.0121
hsa_circ_0087264	chr9	exonic	GNAQ	down	0.0650

### RT-PCR assay for significantly differentially expressed circRNAs and interaction between targeting miRNA

We performed RT-PCR (pre-treatment, n = 43; post-treatment, n = 43). Trends for the 2 circRNAs were consistent with the results of microarray analysis.

**Figure 3:**
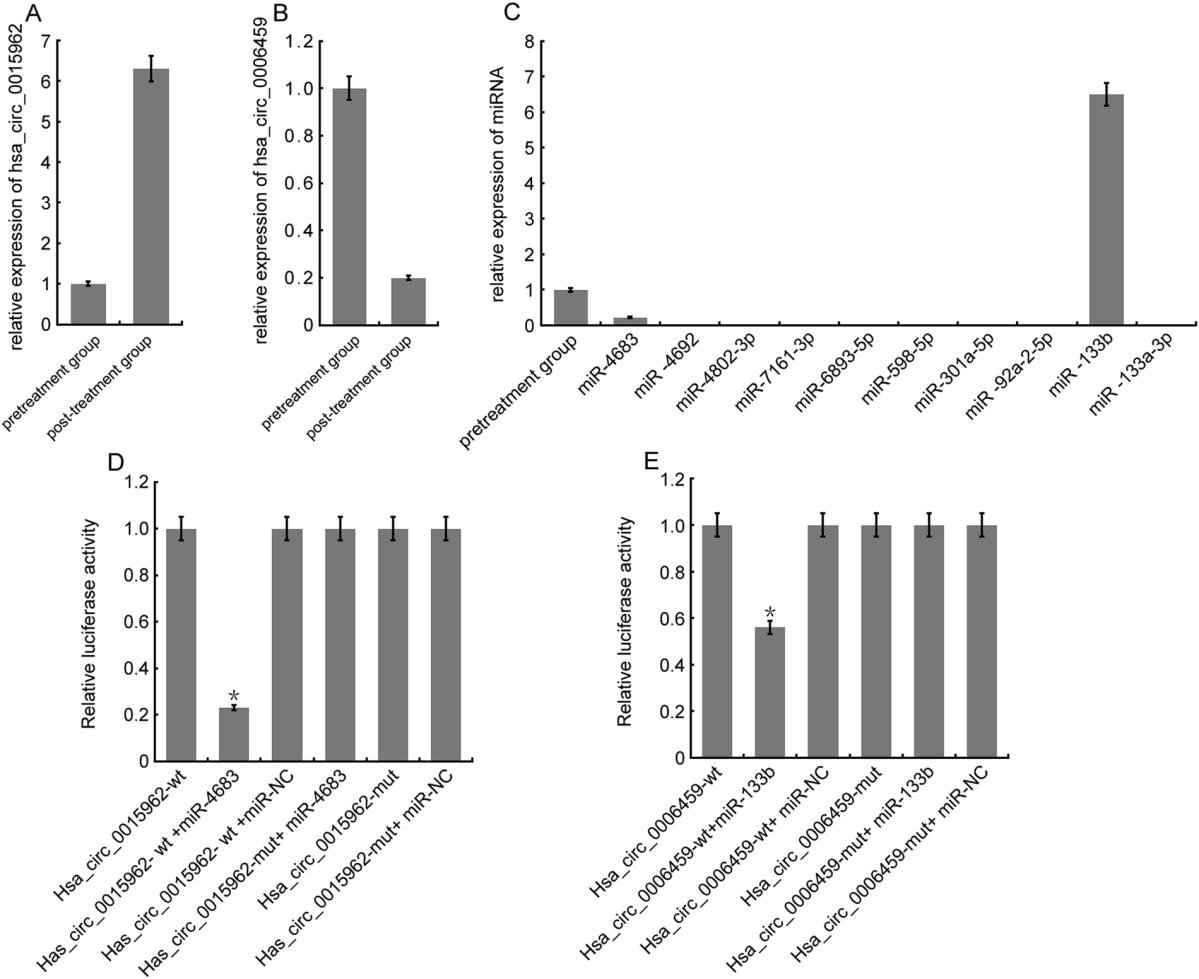
RT-PCR assay for significantly differentially expressed circRNAs and interaction between targeting miRNA. (**A**) Relative expression of has_circ_0015962; (**B**): Relative expression of has_circ_0006459; (**C**) Relative expression of miRNA; (**D**) hsa_circ_0015962-mut and hsa_circ_0015962-WT are respectively co-transfected with miR-4683 mimics into 293T cells. Luciferase activity: ^*^P < 0.05 vs other groups. NC:negative control,miR-NC:a inhibitor of miRNA;mut:mutation. (**E**) hsa_circ_0006459-mut and hsa_circ_0006459-WT are respectively co-transfected with miR-4683 mimics into 293T cells. Luciferase activity: ^*^P < 0.05 vs other groups. NC:negative control,  miR-NC:a inhibitor of miRNA; mut:mutation.

The results indicated that the expression of hsa_circ_0015962 was higher in the posttreatment group than pretreatment group (Fig. 3A). hsa_circ_0006459 expression was lower in the posttreatment group than in the pretreatment group (Fig. 3B). Also, while expression of miR-4683 was lower, miR-133b expression was higher in the posttreatment group than in the pretreatment group. We could not detect the expression of miR-4692, miR-4802-3p, miR-7161-3p, miR-6893-5p, miR-598-5p, miR-301a-5p, miR-92a-2-5p, and miR-133a-3p in the posttreatment samples (Fig. 3C). To investigate the regulatory mechanisms of hsa_circ_0015962 and hsa_circ_0006459 expression, we constructed wild-type and mutant hsa_circ_0015962 and hsa_circ_0006459 vectors, which were respectively co-transfected with miR-133b and miR-4683 mimics into 293T cells. Luciferase activity was detected, and showed that hsa_circ_0015962 binds to miR-4683, and hsa_circ_0006459 binds to miR-133b (Fig. 3D,E). Taken together, these results indicate that hsa_circ_0006459 targets and negatively regulates miR-133b expression, and hsa_circ_0015962 targets and negatively regulates miR-4683 expression.

### Correlation of clinical variables with hsa_circ_0015962 and hsa_circ_0006459 in PBMCs from post-treatment samples

In order to evaluate the correlation between circRNAs differentially expressed in PBMC_S_ of peripheral blood of patients with dengue fever and biological indicators, we analyzed for the Pearson correlation coefficient to assess the correlation between post-treatment clinical data and hsa_circ_0015962, hsa_circ_0006459 (Table 5). The results showed that hsa_circ_0006459 was negatively correlated with WBC, PLT and differential lymphocyte count (r = −0.259, r = −0.356, r = −0.203; *P* < 0.05). The levels of hsa_circ_0015962 in PBMCs from post-treatment samples positively correlated with WBC, PLT, and differential lymphocyte count (r = 0.562, r = 0.789, r = 0.35, *P* < 0.05).

**Table 5:** Pearson correlation coefficient was used to analyze the correlation between hsa_circ_0015962, hsa_circ_0006459 and clinical related variables.

Item	hsa_circ_0015962	hsa_circ_0006459
WBC	0.562^*^	−0.259^*^
PLT	0.789^**^	−0.356^*^
Lymphocyte	0.35^*^	−0.203^*^
Monocyte	0.115	−0.052
Neutrophi	0.08	−0.005

^*^P < 0.05, ^**^p < 0.01.

## Discussion

In our study, we aimed to identify a potential prognostic biomarker in PBMCs of patients with DF. The results showed that hsa_circ_0015962 expression was significantly increased in DF patients, and hsa_circ_0006459 was significantly decreased in DF patients, and therefore can serve as potential prognostic biomarkers for Dengue fever patients. With the development and maturity of high-throughput sequencing technology, more and more circRNAs have been discovered and named, and their biological characteristics, formation mechanism and action mechanism have been revealed gradually^11^. Different from the traditional linear RNA, circRNAs are closed-loop and not easily degraded by nucleic acid exonuclease RNaseR, so they have good stability^12^. Therefore, circRNA can be a potential diagnostic and prognostic biomarker. Hsa_circRNA_103571 may serve as a potential biomarker for diagnosis of active tuberculosis (TB), and hsa_circRNA_103571-miRNA-mRNA interaction may be a novel mechanism in active TB^13^. Gastric Adenocarcinoma-Associated, Positive CD44 Regulator. The intergenic lncRNA GAPLINC may promote primary hepatocellular carcinoma (PHCC) progression and serve as a potential prognostic marker and therapeutic target in PHCC^14^. Salivary circRNAs hsa_circ_0001874 and hsa_circ_0001971 were reported as novel biomarkers for the diagnosis of oral squamous cell carcinoma^15^. In addition, hsa_circ_0074362 probably plays a role in the initiation of gastric cancer and may be a potential biomarker of gastric cancer^16^. Also, hsa_circ_0001785 showed higher expression in peripheral blood of breast cancer patients, and thus could be a stable biomarker for diagnosis and disease progression^17^. The detection of cireRNA mainly focuses on tissue samples. This method is traumatic and not suitable for early clinical diagnosis of tumors. Clinical samples, such as serum, urine and body fluids, are much easier than tissue samples.The expression of circRNA in serum and disease-related body fluids (gastric juice, cerebrospinal fluid, serous effusion, etc.) should be further studied in the future^9^. The characteristics of circRNA (universality, conservativeness, tissue-cell specificity and stability) also suggest that It may be superior to linear RNA in the detection of clinical samples. High diagnostic value^9^.

Upon infection, the virus first proliferates in the capillary endothelial cells and is then released into the bloodstream. The resulting viremia leads to further infection of mononuclear macrophages in the bloodstream and tissues and ultimately causes DF^18^. Leukopenia, especially neutropenia, is the primary laboratory finding; and thrombocytopenia occurs in most cases^6^. In our study, we found that hsa_circ_0006459 was negatively correlated with WBC, PLT, and lymphocyte count. hsa_circ_0015962 levels in PBMCs from post-treatment samples positively correlated with WBC, PLT, and lymphocyte counts. These observations may facilitate future pathophysiological research on treatment response of DF and help to determine whether circRNAs in PBMCs could be used as novel, non-invasive biomarkers for diagnosis and prognosis of patients with DF.

CircRNA plays an important role in various diseases,CircRNA can be used as microRNA sponge, RBP sponge or direct binding Target RNA regulates the expression of RNA^19,20^. In this study, we found that the expression of hsa_circ_0015962 and miR-133b was higher in the posttreatment group than pretreatment group. Expression of hsa_circ_0006459 and miR-4683 was lower in the posttreatment group than in the pretreatment group. Moreover, that hsa_circ_0015962 binds to miR-4683, and hsa_circ_0006459 binds to miR-133b.This study shows that hsa_circ_0006459 targets and negatively regulates miR-133b expression and hsa_circ_0015962 targets and negatively regulates miR-4683 expression, and this provides a new direction for research on the pathological mechanism of treatment response of DF. CircFUT10 regulates myocardial proliferation and differentiation by competitively binding to miR-133a^21^. circHECW2 competitively sponges miR-30D and upregulates ATG5, thereby promoting transformation of cerebral microvascular endothelial cells into stromal cells and consequent damage to the blood-brain barrier^22^. Renal miR-150 showed a tendency to negative correlation with circHLA-C in patients with lupus nephritis. Thus, circHLA-C may play an important role in the pathogenesis of lupus nephritis by sponging miR-150^23^.

There are some limitations in our study. First, dengue fever is a seasonal disease, fewer cases have been collected. Second, we did not confirm the binding between hsa_circ_0015962 and miR-4683 or between hsa_circ_0006459 and miR-133b with RNA pull-down assay. Third, We did not analyze the expression of circRNAs in plasma, serum, saliva and exosomes of patients. Studies have shown that the expression of cirRNA in exosomes is more stably than that in body fluids, and has higher diagnostic value^24^.

In our further study, we will explore the limitations in this study. To the best of our knowledge, this is the first study that used circRNA microarray to determine the expression of circRNA in PBMCs of pre- and post-treatment samples from DF patients. It is expected that the results of our study will provide a theoretical basis for elucidating the pathogenesis of dengue fever and provide a strong basis for guiding the prognosis of dengue fever in clinic.

### Ethics statement

The Ethics Committee of panyu central hospital of Guangzhou approved this study.

## Supplementary information

Supplementary file1

Supplementary file 2

Supplementary file 3
